# Synthesis and Electrical Percolation of Highly Amorphous Polyvinyl Alcohol/Reduced Graphene Oxide Nanocomposite

**DOI:** 10.3390/ma16114060

**Published:** 2023-05-30

**Authors:** Renata Adami, Patrizia Lamberti, Marcello Casa, Nicole D’Avanzo, Eleonora Ponticorvo, Claudia Cirillo, Maria Sarno, Dzmitry Bychanok, Polina Kuzhir, Changjiang Yu, Hesheng Xia, Paolo Ciambelli

**Affiliations:** 1 Department of Physics, University of Salerno, 84084 Fisciano, Italy; 2 Centre NANO_MATES, University of Salerno, 84084 Fisciano, Italy; 3 Department of Information and Electrical Engineering and Applied Mathematics, University of Salerno, 84084 Fisciano, Italy; 4 Narrando Srl, Via Arcangelo Rotunno 43, 84134 Salerno, Italy; 5 Research Institute for Nuclear Problems Belarusian State University, 220030 Minsk, Belarus; 6 Department of Physics and Mathematics, University of Eastern Finland, 80101 Joensuu, Finland; 7 State Key Lab of Polymer Material Engineering, Sichuan University, Chengdu 610065, China; 8 Department of Industrial Engineering, University of Salerno, 84084 Fisciano, Italy

**Keywords:** highly amorphous polyvinyl alcohol, reduced graphene oxide, ‘in situ’ thermal reduction, conductive polymers, percolation threshold

## Abstract

Polyvinyl alcohol is the most commercially water-soluble biodegradable polymer, and it is in use for a wide range of applications. It shows good compatibility with most inorganic/organic fillers, and enhanced composites may be prepared without the need to introduce coupling agents and interfacial modifiers. The patented high amorphous polyvinyl alcohol (HAVOH), commercialized with the trade name G-Polymer, can be easily dispersed in water and melt processed. HAVOH is particularly suitable for extrusion and can be used as a matrix to disperse nanocomposites with different properties. In this work, the optimization of the synthesis and characterization of HAVOH/reduced graphene oxide (rGO) nanocomposite obtained by the solution blending process of HAVOH and Graphene Oxide (GO) water solutions and ‘in situ’ reduction of GO is studied. The produced nanocomposite presents a low percolation threshold (~1.7 wt%) and high electrical conductivity (up to 11 S/m) due to the uniform dispersion in the polymer matrix as a result of the solution blending process and the good reduction level of GO. In consideration of HAVOH processability, the conductivity obtained by using rGO as filler, and the low percolation threshold, the nanocomposite presented here is a good candidate for the 3D printing of a conductive structure.

## 1. Introduction

Conductivity-based polymer composites are a category of materials that respond to the combined demands of conductivity, shielding performance, and printability in lightweight material systems. These particular systems are polymers and composites that conduct and shield metals and, at the same time, keep the adhesive and printable properties of polymers; this allows the creation of a totally new variety of possibilities and opportunities for composite products. Conductive polymer composites have already been used in a wide range of important applications, such as electronic packaging [1], conductive and protective coating [2], sensor [3], EMI (electromagnetic interference) shielding [4], hydrogels [5], and aerospace applications [6]. Semiconductor nanostructured metal oxide has, on the other hand, potential photocatalytic properties and antibacterial activity and can be used against resistant infectious agents and toxic dyes [7,8]. Polymer nanocomposites show different electric, thermal, and mechanical properties depending on the type of nanofiller and the preparation method of the nanocomposites [9]. When the nanofiller is conductive and its content approaches a critical value, namely the percolation threshold, a continuous electrically conductive network is formed by these nanoparticles; therefore, the whole polymer composite becomes electrically conductive. According to the percolation theories, the percolation threshold depends on the filler shape, size, and aspect ratio. In particular, a very high aspect ratio can strongly decrease the percolation threshold of a nanocomposite [10]; nevertheless, the final electrical conductivity is affected by the electrical conductivity of the pure nanofiller. Due to its hydrophilicity, GO is a good nanofiller in hydrophilic and biocompatible polymers, and it is particularly suitable for solution blending method in water, which provides better mixing compared to other methods for composite production [11]; this is mainly due to the lower viscosity of the polymer in solution, as opposed to a melt, coupled with mechanical stirring or ultrasonication that aid in better dispersion of nanoparticles in the polymeric matrix [12]. However, GO does not have good conductivity; therefore, since 2D nanomaterials have become the best choice in the field of nanocomposites, reduced graphene oxide (rGO) has often been used as nanofiller, because it possesses properties similar to graphene but contains oxygen-carrying groups like GO. The mechanical, optical, and conductive properties of rGO are similar to that of graphene due to the presence of defects and oxidized chemical groups, although it has better conductivity. rGO is easily produced by the reduction of GO and can be synthesized in the desired amount very easily. It can be produced using inexpensive graphite as raw material by cost-effective chemical methods with a high yield, and it is highly hydrophilic, forming stable aqueous colloids to facilitate the assembly of macroscopic structures by simple and cheap solution processes, which are important characteristics in the case of large-scale uses of graphene [13]. Recent studies report that GO can be reduced at low temperatures (<300 °C), leading to the production of highly conductive rGO [14].

Among biodegradable polymers, poly(vinyl alcohol) (PVOH) is the most commercially water-soluble polymer in use for a wide range of traditional applications such as papermaking, glue, and textile, as well as for innovative application as smart hydrogels [15], food packaging [16], and fuse deposition modeling [17]. PVOH shows good compatibility with most inorganic and organic fillers, and enhanced composites may be prepared without the need to introduce coupling agents and interfacial modifiers [5,18,19,20]. A high amorphous polyvinyl alcohol (HAVOH), which can be easily dispersed in water and melt processed, has been patented and commercialized with the trade name “G-Polymer”, and it has been studied in a blend with chemically modified organoclays [21]. It is based on polyvinyl alcohol modified with diol monomers and has excellent extrusion processability, water solubility, and oxygen barrier properties; furthermore, it can be easily used for coating, and it is biodegradable [22,23,24]. In the literature, there are some works about composite based on PVOH and GO in the form of light aerogel, with interesting functional properties such as fire resistance and EMI shielding [25] and potential applications in electrodes, sensors, batteries, pressure-sensing applications, and supercapacitors [26]. In particular, the possibility that in the conduction mechanisms also, protons can assume a relevant role together with the electronic charge carriers is explored, opening new routes for the study of conductivity behavior in nanocomposites [26]. However, there are few works about composites based on PVOH and rGO, and the most significant report PVOH-based composites with rGO inclusions, with a very good percolation threshold (~1 wt%) but low conductivity (~10^−3^ S/m), due to the weak interaction between PVOH moieties and reduced graphene oxide functional groups [27,28]. Moreover, to the best of our knowledge, there are no studies reporting HAVOH/rGO (GP_rGO) composites and their electrical behavior above and below the percolation threshold. Herein, in this work, the optimization of a novel process for the production of GP_rGO composites by ‘in situ’ thermal reduction of GO, previously inserted in the HAVOH matrix by solution blending, is reported. The reaction time was optimized with the support of X-ray Diffraction (XRD) analysis results. The composites were prepared by increasing rGO concentration in a range from 1 wt% to 20 wt%. The electrical behavior and mechanical properties were evaluated above and below the percolation threshold. Nevertheless, by considering that the nanocomposites obtained with carbon nanoparticles are promising candidates for electromagnetic (EM) applications, such as shields, filters, and radar absorber materials (RAMs), and that the 3D printing capability should be used to obtain complex and appropriate geometric device shapes, the frequency response of the developed nanocomposites is crucial to evaluate the applicability of the novel material for such kinds of devices. To this aim, not only is DC electrical conductivity used, but its Ka-band behavior is also measured here and reported in order to compare the performance of the different nanocomposites in terms of conductivity at such a frequency range.

## 2. Materials and Methods

High Amorphous Polyvinyl Alcohol (HAVOH, trade name G-Polymer—GP) was purchased from the Nippon Synthetic Chemical Industry Co., Ltd., Osaka, Japan, in form of powder, used primarily for aqueous solution applications. Graphene Oxide (GO, SE2430) was purchased from The Sixth Element Materials Technology Co., Ltd., Changzhou, China. Deionized water, purchased by Fluka Analytical, was used throughout the experiments.

**Figure 1:**
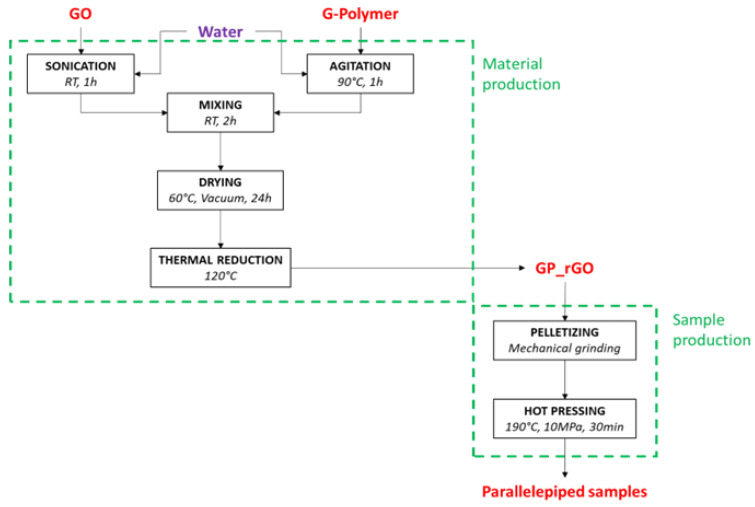
Schematic representation of GP/rGO preparation. RT = room temperature.

The GP/rGO nanocomposites were prepared as reported in the scheme in Figure 1.

**Figure 2:**
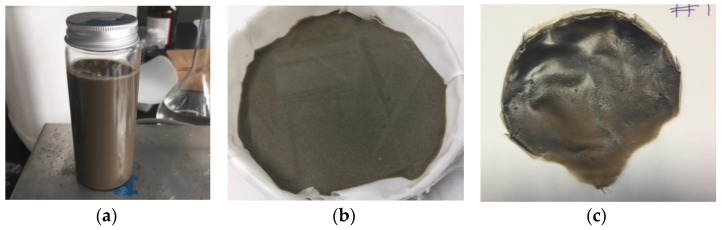
(**a**) Water solution with GO and G-polymer, (**b**) mold-cast solution in a Petri dish, and (**c**) GP/GO nanocomposite after drying at room conditions.

The nanocomposites were prepared by mixing two water solutions containing GO and HAVOH, respectively, in order to obtain the required GO wt% in the polymer (GP_GO). An amount of 4 g of HAVOH was slowly added to water at room temperature, and then the solution was heated up to 90 °C and kept in stirring condition for 1 h; eventually, it was cooled down promptly after completing the dissolution. At the same time, 1 g of GO was dispersed in 100 mL of deionized water by bath sonication at maximum power for 1 h; the bath water was cooled down after the first 30 min of sonication. After both solutions reached room temperature, they were mixed together and kept for 2 h in stirring conditions. The obtained solution was cast in a Petri dish and dried at 60 °C overnight. Then, the physisorbed water was removed by vacuum drying for 24 h, and the weight was monitored. A brownish, flexible compound was obtained. The solid composites obtained underwent thermal treatment for ‘in situ’ GO reduction for different times, 1 h, 3 h, 6 h, 10 h, and 24 h, at 120 °C in atmosphere air. The level of reduction of GO was monitored by X-ray analysis and Raman spectroscopy. In Figure 2, the appearance of the samples is reported.

**Figure 3:**
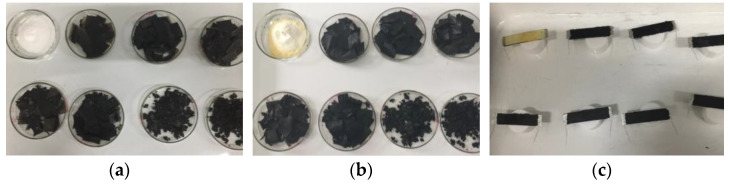
(**a**) Nanocomposites with increasing concentration of GO before the ‘in situ’ reduction, (**b**) nanocomposites with increasing concentration of GO after ‘in situ reduction, and (**c**) samples for electrical measurements.

After the optimization of the reduction time, GP/rGO composites with different concentrations (1, 3, 6, 9, 12, 15, and 20 wt% of GO) were prepared (Figure 3) with the procedure described in the GP/rGO nanocomposite synthesis optimization paragraph, in order to evaluate the electrical conductivity of the materials. For this reason, the materials obtained after ‘in situ’ reduction were mechanically ground and hot pressed to obtain paralepidid samples (size 4 cm × 1 cm × 1 mm, Figure 3c). The hot-press method was optimized in order to obtain solid and compact samples, also at high concentrations of rGO, and to minimize the HAVOH degradation. The standard hot-press procedure was as follows: 5 min at 190 °C, 25 min at 190 °C and 100 bar, 10 min cooling down to room temperature at 100 bar, and depressurization to atmospheric pressure. The electrical conductivity of the composites was measured by a two-probe method.

X-ray Diffraction (XRD) analysis was performed on a Philip-X’Pert X-ray diffractometer fitted with a goniometer detection device (anode 40 kV, filament current 35 mA). Nickel-filtered Cu Kα radiation of wavelength 0.1542 nm was directed at the samples in their through direction. The goniometer scanned diffracted X-rays in the range of 2 θ = 5 to 45° at a scan speed of 0.15°/s.

Raman spectroscopy (LabRAM Aramis, Horiba Jobin–Yvon, Longjumeau, France) with a 532.8 nm laser excitation was used to analyze the synthesized samples. For statistical accuracy, five measurements in parallel were carried out for each sample.

The electrical conductivity of the composites was determined by a two-probe method with a pico-ammeter (Keithley 2400, Keithley Instruments TEKTRONIX, Inc., Beaverton, OR, USA).

The electromagnetic response of the samples was experimentally investigated in the Ka-band (26–37 GHz) using a scalar network analyzer (ELMIKA R2-408R, Vilnius, Lithuania), and measurements were carried out in a 7.2 × 3.4 mm waveguide. The EM properties of the samples were derived by simulation and elaboration using a program in “Phyton” environment. The analytical method and the elaboration program developed were validated by measuring sample of neat HAVOH and a reference sample of Polytetrafluoroethylene (PTFE). The results for the imaginary and real parts of dielectric permittivity were in agreement with each other [29].

The reduced samples were analyzed by SEM (Scanning electron microscopy) (TESCAN- VEGA LMH; 230 V) analysis, with no further coating. Energy dispersive x-ray analysis EDAX FT-IR spectra were obtained by Nicolet iS50 FT-IR.

The thermal characteristics were investigated by thermo-gravimetric and derivative thermo-gravimetric analysis (TG-DTG) with an SDTQ 600 Analyzer (TA Instruments, New Castle, DE, USA); the measurements were performed at 10 K/min heating rate in flowing air.

## 3. Results and Discussion

**Figure 4:**
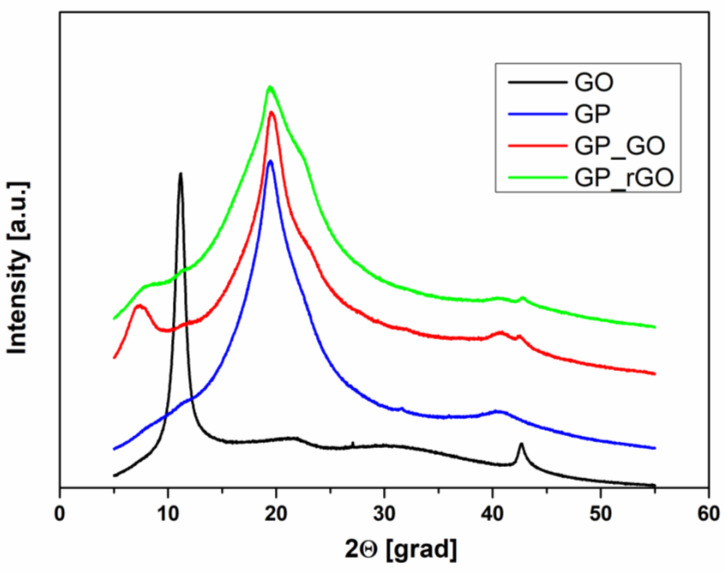
XRD pattern of untreated GP, commercial GO, and the composites GP_GO and GP_rGO (reduced ‘in situ’).

The optimization of the conditions was carried out on the samples at 20 wt% of GO, which is a very high loading percentage in a polymer. In Figure 4, XRD patterns of untreated HAVOH (GP), commercial Graphene Oxide (GO), and the composites GP_GO and GP_rGO (reduced ‘in situ‘) are reported.

As expected, GP shows a semi-crystalline structure, enlightened by the presence of three crystalline peaks in the XRD pattern at approximately 20, 23, and 40 degrees. In the range [10,11,12,13,14,15,16,17,18,19,20,21,22,23,24,25,26,27,28,29,30], the pattern presents the overlapping of two different families of crystals with similar size (α, β); the α-type crystal is six times more present in the polymer matrix, and the large peak at about 40 degrees is related to the amorphousness of the polymer [30,31]. Commercial GO shows two peaks: a more pronounced one at about 11 degrees and a smaller one at about 43 degrees.

It is interesting to notice that in the composites, the typical peak of GO is shifted towards a smaller angle (around 7.8 degrees), meaning that the interplane distance increases due to the presence of the polymer.

**Figure 5:**
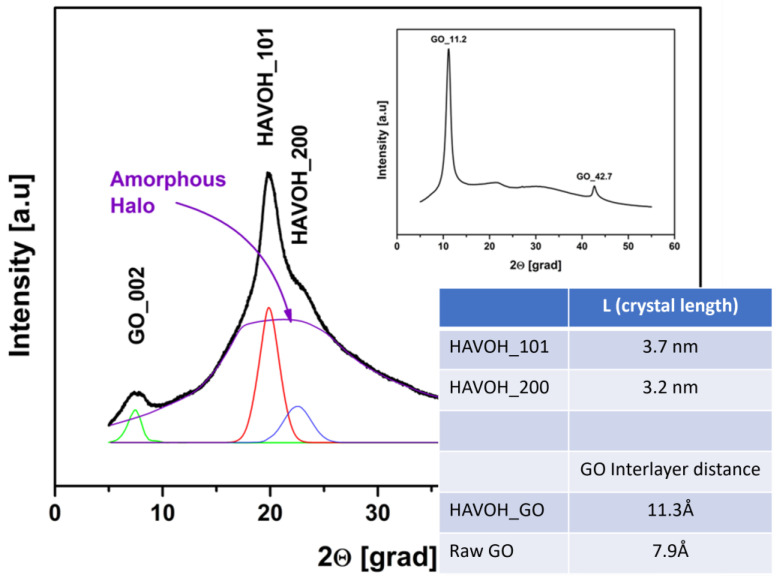
XRD pattern of GP/GO nanocomposite. In the insert commercial GO, pattern is reported. The table shows the crystal length of the polymer and the interlayer distance of raw GO and GO in the polymer matrix.

In Figure 5, XRD patterns of the GP/GO nanocomposite and of the raw GO (in the insert) are reported. The GP peaks were resolved using a deconvolution method with the Lorentz distribution function [32].

The pattern of GP/GO can be reconstructed using four different signals. The GO peaks at around 8 degrees, and two crystalline bands of the polymer, [101] and [200], at around 20 and 23 degrees, respectively. The halo, more intense in the range of 15–30 degrees, was pictured after the subtraction of crystalline peaks obtained by the deconvolution; the halo is typical of the amorphous phase of the PVOH and can be attributed to the amorphous phase of HAVOH. This analysis gives two significant pieces of information by using Bragg’s law and the Sherrer equation: the average crystal length of the crystalline phase of the polymer is around 3.4 nm, and the interlayer distance among the GO planes in the matrix is around 11 Å. Such a distance is around 8 Å in the raw GO, confirming that the shift of the typical peak of GO towards a smaller angle observed in the GP/GO composites is related to an increase in the interlayer distance. It is expected that, during the solution blending process, the polymer chains insert among GO layers, avoiding their aggregation and increasing their distance.

**Figure 6:**
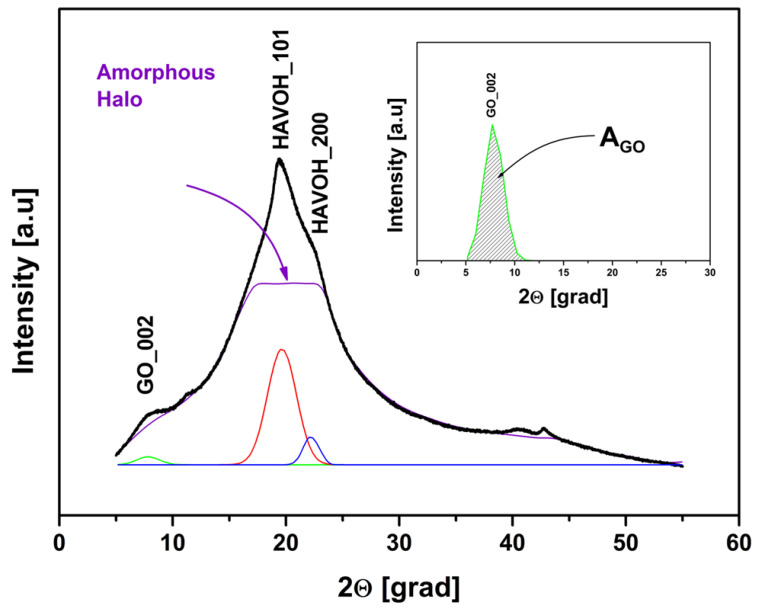
XRD pattern of GP/rGO after 10 h reduction. Area of GO peak in the insert.

The XRD analysis was also used to monitor the GO reduction reaction behavior overtime at 120 °C. It was observed that the GO signal decreases in intensity and area (AGO) over time. Figure 6 shows the XRD pattern of the GP/rGO nanocomposites after 10 h at 120 °C, and the area of the GO signal in the insert. During the ‘in situ’ reduction, the intensity of the GO peak at about 7.78 degrees is decreased, and the morphology of HAVOH is changed: comparing Figure 5 and Figure 6, an increase in the area of the two peaks related to the families of crystals (respectively red and blue lines) can be noted, with the consequent decrease in the amorphous part, which can also be observed in Figure 4.

**Figure 7:**
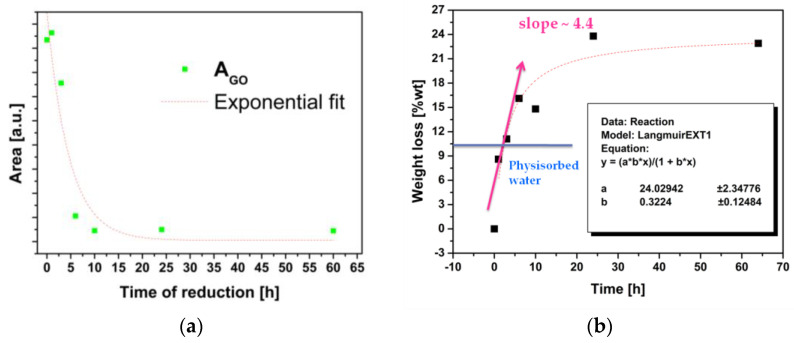
(**a**) The evolution of the underlying area of the GO peak during the reaction and (**b**) the weight loss of the composite during reaction.

AGO was monitored over time and evaluated by normalization of the underlying area of the spectra. In Figure 7a, the area of the typical GO peak versus the time of ‘in situ’ reduction between 1 and 60 h is reported, whereas, in Figure 7b, the weight loss of the samples during the same range of reduction time is shown. The physisorbed water was evaluated by a high vacuum drying process.

As can be observed, the GO in the composite undergoes a reduction process over time when it is exposed to 120 °C, and after less than 5 h of the reduction reaction, the composite already starts to lose the functional group, together with water. Following the AGO evolution during this time, the optimal time of the reduction reaction is found to be 15 h.

**Figure 8:**
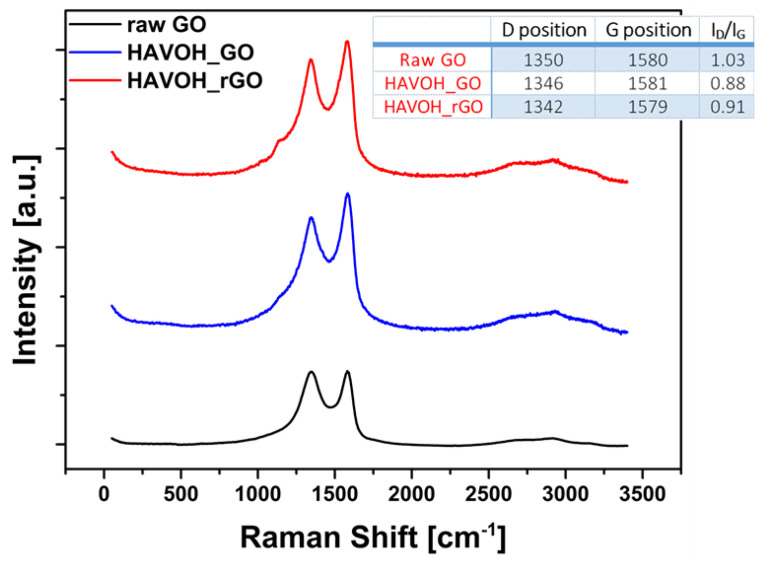
Raman spectra of raw GO, GP/GO, and GP/rGO nanocomposites.

The Raman spectra of raw GO, GP/GO, and GP/rGO composites (Figure 8) show that the shape of the spectra is similar for all the materials, though a slight blue shift of the D band position after the reduction is observed; it could be attributed by electron doping of the rGO surfaces, as reported by Liu et al. [33]. The increase in the ID/IG ratio can be attributed to the decrease in the distance between the defects along the rGO surface [34]. The values of the positions of the D and G bands and their intensity ratio for the three species are reported in the insert table in the figure. Both these phenomena are promising for the production of a conductive nanocomposite.

**Figure 9:**
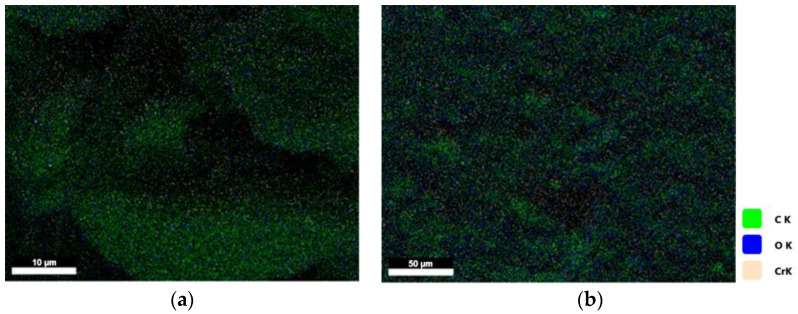
EDAX analysis of sample GP (**a**) and GP_rGO 15 wt%) (**b**).

After the reaction time was optimized and the reduction was successful, nanocomposites with different concentrations of GO were prepared by the solution blending method, namely 1, 3, 6, 9, 12, 15, and 20 wt%. The samples were dried and treated at 120 °C for 15 h, and it was observed from EDAX investigations that the elemental analysis appears to be the same for neat HAVOH and nanocomposite GP_rGO (Figure 9); therefore, no impurities were introduced during the procedure.

**Figure 10:**
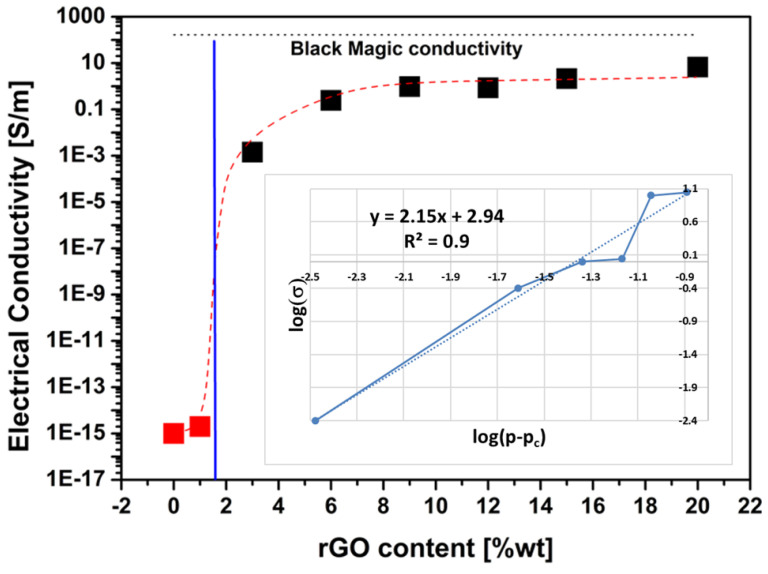
Percolation curve for GP/rGO nanocomposite in DC (inset percolation curve parameters’ detection).

Samples were hot pressed, and their electrical conductivity was measured in order to evaluate the percolation threshold and the behavior of the nanocomposite above and below it. The sample thickness was about 0.65 ± 0.23 mm as a result of the hot pressing. In Figure 10, the evolution of the electrical conductivity with the variation of rGO concentration is reported. As expected, the percolation threshold for GP/rGO nanocomposites is situated around 1 and 2 wt%, while less expected is that at 9 wt% of rGO, the conductivity of the nanocomposite is already 1 S/m, three orders of magnitude higher than the reported value of the PVOH/rGO system and not far from the conductivity of the commercial product Black Magic filament, which is a system composed by poly lactic acid, graphene, and carbon nanotubes. This is a very interesting result, since the electrical conductivity of the commercial benchmarks is mainly related to the presence of carbon nanotubes in the nanostructure, which are highly conductive [35]. Increasing the rGO content, the plateau is reached (Figure 10), with a conductivity of 11.1 S/m at 20 wt% rGO content and 10 S/m at 15 wt% rGO content already, higher than the commercial benchmarks. This suggests that a lower amount of rGO can be used with very good performances, which is interesting for commercial applications since less expensive material could be used.

The obtained low percolation threshold and high electrical conductivity can be attributed to the uniform dispersion in the polymer matrix allowed by the solution blending process and a good reduction level of GO. As a matter of fact, the inset in Figure 10 on the DC conductivity measurements shows the curve used to derive the parameters governing the conductivity of the composites according to the classical percolation theory [36] as in the following:

(1) $$\sigma =\sigma_{0}{\left(p-p_{c}\right)}^{t}$$

where *p* is the relative volume fraction of graphene and *p_c_* is the percolation volume fraction or percolation threshold, while *σ* and *σ*_0_ are the conductivity of the composite and the nano-inclusion, respectively. Furthermore, *t* is the critical power law exponent and depends on the dimensionality of the electrical network that is established in the material due to the percolation paths. In Table 1, the obtained parameters are reported. 

With a statistical R^2^ equal to 0.97, from the interpolated curve, a percolation threshold of 1.7 vol% is obtained, corresponding to 2.5 wt% if 1.9 g/cm^3^ [37] and 1.3 g/cm^3^ [38], which are considered as the densities for rGO nanoparticles and HAVOH matrix, respectively. The same graph helps to estimate a nano-inclusion conductivity of 871 S/m, being log(σ_0_) = 2.94 as the intercept of the interpolating line in the inset shown. Moreover, the 2.15 value for the critical exponent *t* obtained here indicates the creation of a three-dimensional system, i.e., a 3D electrical network composed of the percolation paths supported by the uniform distribution of the nanofillers [39].

**Figure 11:**
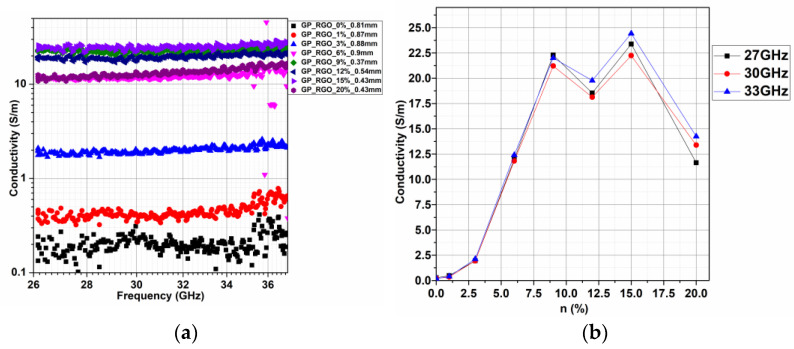
(**a**) Conductivity of GP/rGO samples in the range 26–37 GHz and (**b**) conductivity vs. percentage of rGO at different values of Frequency (GHz).

The electrical conductivity of GP/rGO samples was also measured in the Ka-band 26–37 GHz, and the results are reported in Figure 11a,b, showing the conductivity of different samples varying the frequency (GHz) and the variation of GO content at fixed frequencies, respectively. The membranes were mechanically reduced in size in order to fit in the waveguide; when necessary, their thickness was reduced in order to have a thickness of 0.65 ± 0.23 mm. The elaboration of the results took into account the thickness information. The imaginary and the real part of conductivity were constant with the increase in frequency; in particular, for the real part, an increment of the value of conductivity with the amount of the percentage of rGO up to 9 wt% was observed (Figure 11a). For higher percentages of rGO content, a random response was shown, probably for the degradation of samples at a high percentage of graphene filler. An interesting electrical conductivity is obtained at an rGO content of 15 wt%.

It is well known that the frequency behavior of nanocomposite conductivity above the percolation threshold is constant up to a characteristic frequency, such as in the case of a conductive material due to the predominant resistive behavior governed by the conductive filler [40,41]. In particular, this constant value is maintained up to a characteristic frequency whose value increases at higher filler content. After this frequency, the AC conductivity starts to have an increasing frequency-dependent phase due to the capacitive effect of the electrical percolative network at a higher frequency. Nevertheless, if the AC conductivity of nanocomposites above the percolation threshold is considered, it is possible to observe a typical insulator material behavior corresponding to a fast-increasing value starting from very low frequencies, due to the predominant matrix capacitive effect. As a matter of fact, by observing the Ka-band measurements reported here, it should be considered that the post-percolative sample with 3 wt% already experienced its characteristic frequency, reaching a three-fold higher value of 2 S/m with respect to few mS/m in DC, whereas the other nanocomposites above the percolation threshold are still in a constant phase behavior. Therefore, it is possible to consider the usage of nanocomposites above 3 wt% as a filament to make a Ka-band RAM device, whereas 3 wt% nanocomposites are to be avoided if “conductor” components are of interest.

It can be observed (Figure 11b) that the electrical conductivity values are comparable at the different high frequencies investigated, and there is an unusual behavior at high rGO content. This could be related to morphological problems due to the high rGO concentration in the polymeric matrix.

**Figure 12:**
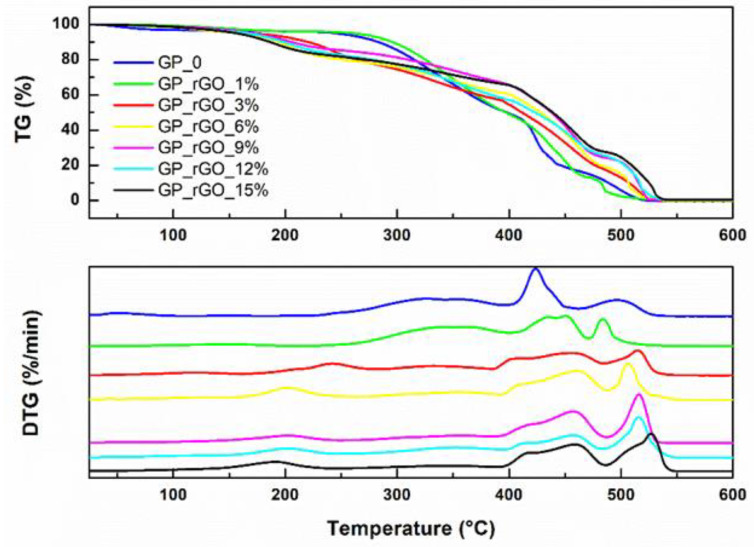
TG-DTG analyses at different rGO content.

Thermogravimetric analyses (Figure 12) report the decomposition phenomena by the increase in temperatures: at <100 °C, there is water elimination; at 100–360 °C, the removal of oxygen functional groups takes place; and at 360–1000 °C, the oxidative pyrolysis of carbon framework can be observed [42].

All the samples do not show any water content, though the temperature at which the thermal phenomena take place varies with the rGO content. The neat HAVOH shows only two thermal phenomena between 400 °C and 550 °C; similar behavior is observed for 1 wt% rGO content. Increasing rGO percentage, several thermal phenomena take place, mainly due to the interaction between the rGO homogeneously distributed in the HAVOH matrix. In particular, the earliest carbon combustion (Tmax = 550–616 °C) moves to higher temperatures. This suggests that the presence of aggregates with the increase in rGO content can influence the structure and morphology of the polymer matrix. Since the distinguished Tmax can be attributed to the maximum of the external heat energy required to overcome the strong bonding within their carbon lattice structure, it can be supposed that the increase in rGO content improves the strength of the bonds.

**Figure 13:**
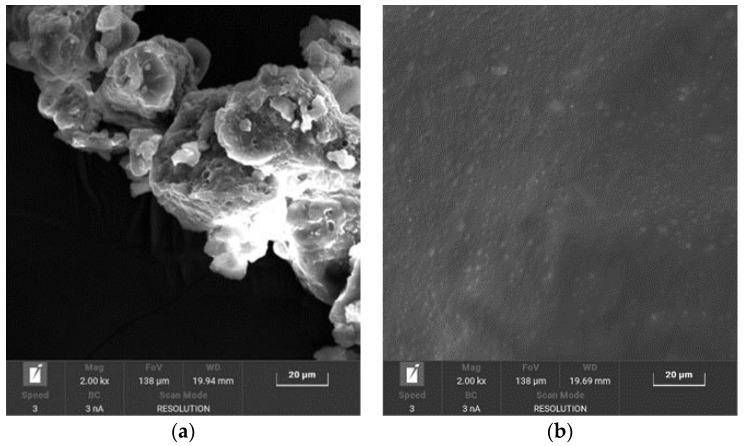

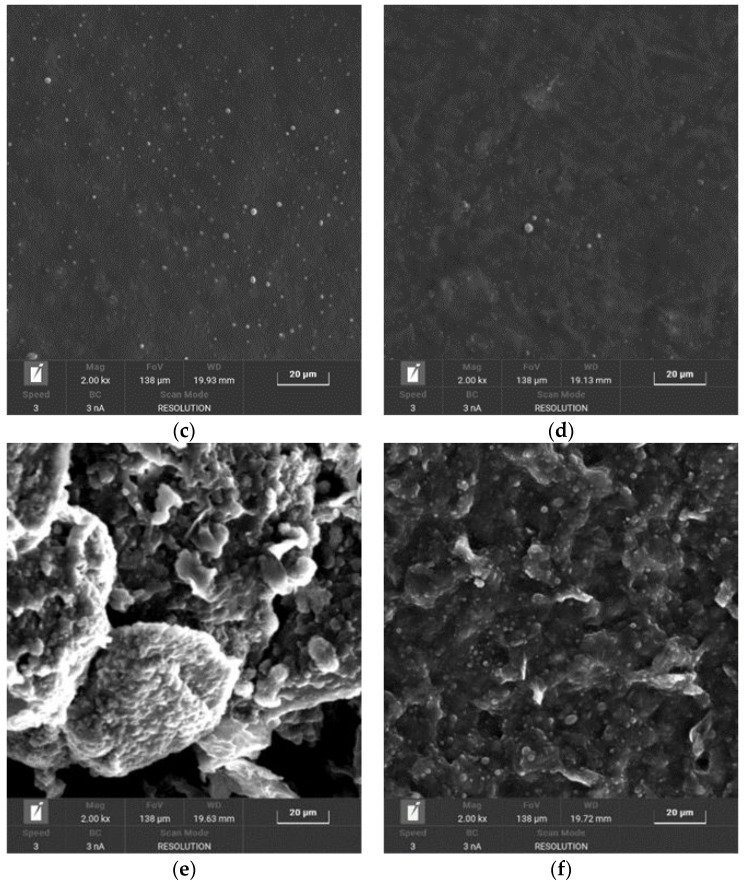
SEM photomicrographs of GP_rGO samples at different rGO content: (**a**) 0 wt%, (**b**) 1 wt%, (**c**) 3 wt%, (**d**) 6 wt%, (**e**) 9 wt%, and (**f**) 12 wt%.

SEM analysis confirmed a change in the morphology of the polymeric structure, increasing the rGO content. The structure with low rGO content appears to be more compact, with a smooth surface. With increasing rGO percentage, the structure is less homogenous, and more fractures can be observed on the sample surface (Figure 13).

SEM structure analysis could explain the differences in the thermal behavior of the samples and in the electrical conductivity. It might be that the rGO concentrations higher than 10 wt% damage the HAVOH structure, with consequences on the nanocomposite characteristics.

## 4. Conclusions

The production of HAVOH and reduced graphene oxide nanocomposite can be successfully obtained using a simple and fast process, which is the solution blending process of HAVOH and Graphene Oxide water solutions and a subsequent ‘in situ’ reduction of GO. The obtained nanocomposite presents a low percolation threshold of about ~1.7 wt% and high electrical conductivity of about 10 S/m when the content of rGO is higher than 15 wt%. These values are due to the uniform dispersion in the polymer matrix allowed by the solution blending process and the good reduction level of GO. 

The composites above the percolation are very conductive, and the structural properties, typical of the HAVOH, are almost retained. A concentration of the filler higher than 9 wt% could raise some morphological and structural problems, with not much increase in the electrical properties. This suggests that a compromise between the performances of the nanocomposite and the amount of the material used as the filler could be considered, with the advantage of the costs of the used material.

In conclusion, GP/rGO composites can be considered commercially competitive compared to benchmarks in several fields, such as electrical conductive composite materials, electromagnetic interference shielding, and additive manufacturing.

Taking into account the processability of the nanocomposites, the conductivity obtained by using rGO as filler, and the low percolation threshold, the nanocomposite presented in this work is a good candidate for the 3D printing of conductive structures for packaging and electromagnetic shielding. Finally, it is worth mentioning that GP/GO composite shows good electrical conductivity already after the hot pressing step (30 min at 190 °C), suggesting the opportunity to develop an ‘in situ’ reductive filament extrusion, obtaining a conductive filament during the extrusion, without the thermal treatment step.

## Figures and Tables

**Table 1:** Percolation curve parameters.

*p_c_* Vol%	*σ*_0_	*t*	R^2^
1.74	871 S/m	2.15	0.97

## Data Availability

The data presented in this study are available on request from the corresponding author.
